# On Bubo

**Published:** 1838-04-01

**Authors:** 


### ON BUBO.

I. Practical Observations on the Venereal Disease and on the Use of Mercury. By *Abraham Colles*, M.D., one of the Surgeons of Doctor Steevens's Hospital, and lately Professor of Surgery in the Royal College of Surgeons, in Ireland. 8vo. pp. 351, 1837.

II. A Treatise on the Venereal Disease and its Varieties. By *William Wallace*, M. R. I. A. &c. &c. &c. 8vo. pp. 382, London, 1833.

III. The Works of John Hunter, F.R.S., with Notes. Edited by *James F. Palmer*, Senior Surgeon to the St. George's and St. James's Dispensary. Vol. II. London, 1835.

In the 53rd number of this Journal we took up the subject of Primary Venereal Sores, at least of some forms of them; and in the 54th, the number preceding the present, we devoted some pages to the consideration of the best method of administering mercury in syphilis. We stated that, from time to time, we should dedicate an article to one of the many forms which venereal maladies assume, and to the useful object of giving our less experienced readers some practical hints upon their management. We select for notice now a not uncommon and a troublesome venereal symptom—Bubo.

The authors, whose remarks will form our text, and the titles of whose works have introduced these observations, have all treated of this symptom, with more or less of circumstantiality. One, Mr. Wallace, has lately fallen a victim to Typhus, a malady which has visited the profession heavily. Although we disagreed with Mr. Wallace on some speculative and practical grounds, we must say that he was possessed of great ingenuity, and distinguished by remarkable industry. He cannot but be deemed a loss to science, and his premature death is much to be regretted.

Perhaps, the most convenient, if not the most natural method of proceeding, will be to examine in detail—the circumstances under which bubo occurs—the various forms that it assumes, and the course that it pursues—the contagious or non-contagious properties of its discharge—its treatment—and, finally, some matters connected with it which do not admit of such definite arrangement. In pursuing this plan we shall probably be obliged to dodge from one author to another, and perhaps it will not always be easy to distinguish our own observations from theirs. But this is trivial, in comparison with the object which we have in view—that of laying a connected and a practical account of this venereal symptom before the readers of this Journal.

### 1. Circumstances under which Bubo occurs.

A. Bubo being essentially an inflammatory enlargement of the inguinal absorbent glands, may result of course, like other inflammations of absorbent glands, from the extension of inflammation of their inferent vessels. Common inflammation of such vessels at their origin or in their course may therefore be, and often is, a cause of bubo.

It is a matter of great practical moment to distinguish buboes of this simple character, from such as arise from the absorption of venereal poison, and must consequently be regarded as a distinct venereal symptom. But, when we turn to the authors before us, we do not find the satisfactory data we require for drawing a distinction between these affections.

Dr. Colles says little, we might call it nothing, on the subject. Mr. Wallace copies John Hunter, and Hunter himself advances opinions which, we believe to be erroneous, if not mischievous.

It is not an uncommon circumstance for the inflammation of gonorrhœa to extend from the urethral mucous membrane, to the integument of the prepuce, and to the loose subcutaneous cellular membrane. The prepuce becomes a little swollen, and rather red. From the inflamed part, a small hard cord may often be felt beneath the skin on the dorsum of the penis, stretching to the pubes, and occasionally leading to an enlarged and inflamed gland there. We need hardly say that in such a case, it is an inflamed absorbent that constitutes the cord, and that the extension of the inflammation has occasioned the enlargement of the gland. The integument usually presents a more or less distinct red streak in the course of the absorbent, as it does in the case of inflamed absorbents elsewhere. Sometimes, too, the inflammation extending from the absorbent to the surrounding cellular membrane, and, exceeding certain limits in intensity, suppuration in that cellular membrane, and one or more abscesses are the result. This, too, is what frequently occurs in inflammation of the absorbents, and need not occasion surprise.

Inflammation of the absorbents of the penis, is one of the most ordinary complications of inflammatory gonorrhœa. We see it much more frequently than inflammation of the testicle or bladder, and as its occurrence admits of an easy explanation, so it seems to be a very natural result of inflammation of the urethral mucous membrane, however that may have originated. We have always regarded this affection as simply an inflammatory one, have treated it in correspondence with such a view, and in no instance have we seen such secondary symptoms as would have shown that it depended on the absorption of a poison. On this point we can speak most positively. In the few cases of unequivocal secondary symptoms after gonorrhœa, which have fallen under our own observation, it has so happened that there has been *no* affection of either the absorbent vessels or glands.

We will now turn to Mr. Hunter. "The second mode of absorption," he says, "is more frequent than the former," (he had been talking of absorption without breach of surface or gonorrhœa,) "and it is when the matter applied has produced a gonorrhœa; and it may happen while the complaint is going on, either under a cure or not. Some of the matter secreted by the inflamed surfaces having been absorbed and carried into the circulation, produces the same complaints as in the former case, by which means the person gives himself the lues venerea."

It will be noticed that Mr. Hunter speaks of the absorption of venereal matter from a gonorrhœa, as a comparatively frequent cause of secondary symptoms. Yet we know that it is not. But to proceed. After observing that inflammation of the absorbent vessels or glands may be a consequence of chancres on the glans or prepuce, he goes on to state that:—

"Sometimes they arise from the thickening of the prepuce in gonorrhœas, that part in such cases being generally in a state of excoriation, as was described when I treated of that form of the disease. These chords often terminate insensibly on the penis, near its root, or near the pubes; at other times they extend further, passing to a lymphatic gland in the groin: this chord can be easily pinched up between the finger and thumb, and it often gives a thickness to the prepuce, making it so stiff at this part as to make the inversion of it difficult, if not impossible, producing a kind of phimosis.

I think I have observed this appearance to arise as frequently from the gonorrhœa, when attended with the before-mentioned inflammation and tumefaction of the prepuce, as from chancres; *which, if my observation is just, is not easily accounted for.* I have observed that absorption is more common to ulcers than inflamed surfaces, or at least the formation of a bubo in the gland, and its effects in the constitution, are more common from an ulcer; but it may be remarked, that the inside of the prepuce, from whence this chord appears to arise, is in an excoriated state. It is possible that this effect may arise from the lymphatics sympathizing with the inflammation of the urethra, but I believe the affection is truly venereal; or it is possible that even the absorption of the coagulable lymph, which was produced from the venereal inflammation, and which is the cause of the tumefaction, may have the power of contamination, as appears to be the case in the cancer.

The thickening, or the formation of this hard chord, probably arises from the thickening of the coats of the absorbents, joined with the extravasation of coagulable lymph, thrown in upon its inner surface, as in inflamed veins.

This chord often inflames so much as to suppurate, and sometimes in more places than one, forming one, two or three buboes, or small abscesses in the body of the penis. When this is going on we find in some parts of this chord a circumscribed hardness; then suppuration takes place in the centre, the skin begins to inflame, the matter comes nearer to it, and the abscess opens like any other abscess.

I have seen a chain of these buboes, or little abscesses, along the upper part of the penis, through its whole length.

This may be supposed to be exactly similar to the inflammation and suppuration of a vein after being wounded and exposed.

Inflammation of the glands is much more frequent than the former, and arises from the venereal matter being carried on to the lymphatic glands, the structure of which appears to be no more than the ramifications and reunion of the absorbent vessels, by which means they form these bodies.

From this structure we may reasonably suppose that the fluid absorbed is in some measure detained in these bodies, and thereby has a greater opportunity of communicating the disease to them than to the distinct vessels, where its course is perhaps more rapid, which may account for the glands being more frequently contaminated." 359.

It is strange, or it may seem so, that a man like Mr. Hunter should overlook an obvious explanation attended with no difficulties, to grasp a remote one confused by contradictions. But, with Mr. Hunter, this was frequently the case, and we agree with one of his able editors, that the powers of his logic were not of a high kind. He found, to revert to the case before us, that, as a general law, ulcerating surfaces were more calculated for absorption than unbroken ones. On the other hand, he found that inflammation of the absorbents arose as frequently from inflammation of the prepuce in gonorrhœa, as from chancre. An ordinary reasoner would have suspected that the cause *was* the inflammation. That appears the direct and simple inference. But Mr. Hunter did not draw it, and he was evidently puzzled.

The enlargement of the lymphatic glands in gonorrhœa, is not always attended with appreciable inflammation of the absorbent vessels of the penis, nor is it limited to the pubic region. Inguinal buboes, occasionally, but, if we may judge from our own experience, not very frequently, ensue."

It will be remarked, that Mr. Hunter speaks of a "chain of buboes" along the whole length of the penis on its upper part. If we define bubo to be enlargement of an absorbent gland, then the application of the term to abscesses of the penis is obviously incorrect. Those abscesses are seated in the loose cellular membrane of the penis, and arise from the extension of the inflammation of the absorbent vessel lying in it.

B. Bubo may occur in gonorrhœa independently of appreciable inflammation or enlargement of the vessels leading to the gland. This form of bubo is usually known by the name of "sympathetic." The term would imply that the extension of inflammation, as well as the absorption of irritating matter, have nothing to do with the production of the bubo. Yet this is by no means certain. It is not uncommon for lymphatic glands to enlarge in other parts of the body, in consequence of irritation in the course of their inferent vessels, without our being able to distinguish enlargement or inflammation of the latter. Are we to suppose that, in cases of this sort, the glandular inflammation is "sympathetic," or that it results from a slight amount of extension of inflammation, or from the direct conveyance of irritating matter to the gland? This is a question very difficult of determination, and our opinions on the subject of "sympathy" are too vague, to lead us to expect its speedy solution. But, at all events, the term "sympathetic bubo" is greatly misapplied, and sometimes syphilitic, more frequently inflammatory buboes, receive this indefinite, if not erroneous, appellation,

If what we have observed is founded in truth the attempt to distinguish sympathetic from other buboes is futile. It would be better in our opinion to abandon the term "sympathetic" in its application to bubo, and to distinguish the latter as inflammatory or specific—as the result of simple irritation and inflammation, or of the absorption of a specific poison.*

---

* As a sample of the confusion that arises from the use of the term sympathetic, or rather from the vague ideas of which that term is the exponent, we may quote a few passages from the work of Mr. Wallace.

"When a bubo occurs in connection with, or has followed immediately after a primary symptom, it is called by some writers a secondary or consecutive bubo.

Secondary buboes, which are to be distinguished from constitutional buboes, or those depending on contamination of the system, may arise either from absorption of the virus, or from sympathetic irritation. The former are called symptomatic, and the latter sympathetic buboes.

This last distinction, though founded in nature, is not of much practical importance, as we have no means of distinguishing with certainty the sympathetic from the symptomatic bubo. It may however be said, that a bubo following catarrhal syphilis, is more generally sympathetic than symptomatic; and that a bubo is most probably sympathetic when it commences in the very earliest stage of a primary symptom, but particularly when this symptom is attended by much irritation or diffused inflammation. Whereas, if a bubo does not occur until the primary disease has been many days, or perhaps weeks in existence, and when c. Bubo from the absorption of a specific poison, may ensue either from gonorrhœa or from syphilis. Whatever may be his abstract opinions on the nature of gonorrhœa, it is impossible for any man of candour and experience to deny, that it is occasionally succeeded by secondary symptoms. They constitute, it is true, the exception not the rule, but that exception is sufficiently frequent to become an object of importance. The occurrence of secondary symptoms proves, if our ordinary notions are correct, the absorption of a special poison. If our notions are correct, too, that absorption will probably take place through the medium of the lymphatics. It is consistent with reason to suppose that in such circumstances the glands on those lymphatics may be irritated, inflamed, enlarged: that, bubo, in short, may be the consequence. Yet, so far as we have seen, this deduction of reason has not been borne out in fact, and in the limited number of cases of secondary symptoms from gonorrhœa which have fallen under our own observation, bubo has not existed.

Syphilitic bubo is more frequent and of more consequence, and it is from this that the common ideas of the nature, the history, and the treatment of bubo would seem to have been essentially derived. It merits then all the attention we can give to it. We shall chiefly follow Mr. Hunter, for succeeding authors have copied his descriptions, as he probably did some of those of preceding ones.

The *time* at which a syphilitic bubo occurs is very various and very indefinite. It is not uncommon for bubo to constitute an early symptom. In the majority of such instances as have occurred to us, the bubo has not attained much magnitude, and has gradually subsided with the subsequent progress of the case. In other cases the bubo has advanced, attained large dimensions, and suppurated. A not uncommon period for bubo to present itself is from the tenth to the twentieth day of the sore. The latter, perhaps, hangs fire, and the stage of granulation, or of cicatrisation is indolent. Nay, the sore may wear an irritable aspect, and appear disposed to spread. Under these circumstances bubo may occur, and, when it does so, may be expected to be troublesome. Mr. Hunter makes few remarks on this subject. The following passage comprises most of what he says.

"It would appear in some cases that it is some time after the absorption of the venereal matter before it produces its effects upon the glands; in some it has

---

neither the primary symptom nor the bubo are attended by much irritation or inflammation, we may, without any great fear of error, conclude that it is symptomatic. It is however to be remembered, that a bubo which was in the first instance sympathetic, may afterwards become symptomatic."

The absurdity of bandying the terms "sympathetic" and "symptomatic" in this way must be obvious. Strictly speaking, every bubo is both sympathetic and symptomatic, and no bubo is singly the one or the other. Why then use terms which, if they have a definite meaning at all, have an erroneous one, and if they have no definite meaning, must mislead? The mass of mankind are governed by sounds. Names, which are but the representatives of things, become in the eye of ignorance, the things themselves, and thus error is generated and diffused. No one acquainted with the history of delusion would ask :—"What's in a name?" The records of human crime and weakness, prove that there is much in it.

been six days at least. This could only be known by the chancres being healed six days before the bubo began to appear; and in such cases it is more than probable that the matter had been absorbed a much longer time before, for *the last matter of a chancre most probably is not venereal;* and indeed it is natural to suppose that the poison may be as long before it produces an action on the parts, when applied in this way, as it is either in the urethra or in forming a chancre, which I have shown to be sometimes six or seven weeks." 360.

It is certainly rare for bubo to occur, after the cicatrization of a venereal sore in a patient treated with mercury. It is so rare, that we consider the patient in these circumstances, as virtually secure from it. This, at least, is the conclusion at which we have arrived from what we have actually seen.

The *situation* of a syphilitic bubo has been much insisted on by some persons. We have heard a surgeon of eminence dwell on this, and insist that syphilitic bubo occurs upon or above the ligament of Poupart, while bubo from irritation of the absorbents of the leg and thigh is seated lower down. This has not corresponded with our own observation, and it has appeared to us that the site of the bubo, from venereal sores upon the penis, is not limited to a particular portion of the groin. Mr. Hunter remarks:—

"The situation of buboes arising from the venereal disease in the penis is, in men, in the absorbent glands of the groin; if a gonorrhœa is the cause of a bubo, one groin is not exempted more than the other; both may be affected: but if a bubo arises in consequence of a chancre, then the groin may be generally determined by the seat of the chancre, for if the chancre is on one side of the penis, then the bubo will commonly be on that side: however, this is not universally the case; for I have known instances, although but few, where a chancre on one side of the prepuce or penis has been the cause of a bubo on the opposite side, which, if arising from that chancre, is a proof that the absorbents either anastomose, or decussate each other. If the chancre be on the frænum, or on the middle of the penis, between the two sides, then it is uncertain which side will be affected.

The situation of the glands of the groin is not always the same, and therefore the course of the absorbent vessels will vary accordingly. I have seen a venereal bubo, which arose from a chancre on the penis, a considerable way down the thigh; on the contrary, I have seen it often as high as the lower part of the belly, before Poupart's ligament, and sometimes near the pubes, all of which three situations may lead to some variations in the method of cure; therefore it may be proper to attend to them.

As the disease most commonly arises from copulation, the situation of buboes is generally in the groin; but as no part of the body, under certain circumstances, is exempt from this disease, we find the nearest external glands between the part of absorption and the heart, everywhere in the body share the same fate with those of the groin, especially if external." 362.

We do not know why the lymphatic glands are more prone to inflame from the passage of the venereal poison than the vessels which carried it to them. We shall not mention the hypotheses that have been framed, because they are only hypotheses. But it is uncommon for any but the glands nearest to the seat of absorption to become affected. And, whether they are or are not, the secondary or ternary series of glands, placed on the lymphatic vessels nearer the centre of the system are seldom, if ever, implicated.

"We never find," says Mr. Hunter, "the lymphatic vessels or glands that are second in order, affected as those along the iliac vessels or back; and I have also seen when the disease has been contracted by a sore, or cut upon the finger, the bubo come on a little above the bend of the arm, upon the inside of the biceps muscle; and in such where the bubo has come in that part, none has formed in the armpit, which is the most common place for the glands to be affected by absorption.

But this is not universal, although common, for I was informed by a gentleman who contracted the disease in the before-mentioned way, that he had buboes both on the inside of the biceps muscle and in the armpit. Another case of this kind I have heard of since; why it is not more common is perhaps not easily explained.

It might be supposed that the matter was weakened, or much diluted by the absorptions from other parts by the time it gets through these nearest ramifications, and therefore has not power to contaminate those which are beyond them; but it is most probable that there are other reasons for this. I once suspected that the nature of the poison was altered in these glands as it passed through them, which was the reason why it did not contaminate the second or third series of glands; and also why it did not affect the constitution in the same way as it did the parts to which it was first applied; but this explanation will not account for the next order of glands to suppurating buboes not being affected by the absorption of venereal matter. It appears to me that the internal situation of the other glands prevents the venereal irritation from taking place in them; and this opinion is strengthened by observing when one of these external glands suppurates and forms a bubo, which is to be considered as a large venereal sore or chancre, that the absorption from it, which must be great, does not contaminate the lymphatics or glands next in order, by the venereal matter going directly through them.

If this be true, then the skin would seem to be the cause of the susceptibility of the absorbents to receive the irritation. Whether the skin has the power inherent in itself, or acquires it from some other circumstance, as air, cold, or sense of touch, is not easily ascertained; but whichever it be, it shows that the venereal matter of itself is not capable of irritating, and that it requires a second principle to complete its full effect, that is, a combination of the nature of the poison and the influence of the skin, and that influence must be by sympathy, and therefore weaker than if acting in the same part, that is, the skin itself, which perhaps is the reason why the venereal matter does not always affect those vessels and glands, while it always does the skin, if inserted into it." 361.

The foregoing quotation offers a fair instance of Mr. Hunter's frequent mode of handling a difficult or a dubious subject. Facts, reasoning, assumptions are all mixed together, and it would seem to have been an inherent mental vice not to distinguish and separate them. Where a fact seems to lead to a theory, he seldom misses the opportunity of theorizing—where a theory does not present itself so naturally, he very frequently smuggles or squeezes it in—and where a fact contradicts his theory, there is more than a solitary instance of his resolutely denying the possibility of the fact altogether.

The latter part of the passage we have quoted is a strange jumble of words, and not a very clear association of ideas. The fact he struggles so vainly to express is a simple one—the syphilitic poison, like the variolous, evinces a predilection to affect the cutaneous system. But it is by no means clear that Mr. Hunter is justified in the proposition he advances:—that "the venereal matter always affects the skin, if inserted into it." There can be no doubt that persons have inoculated themselves with venereal matter, and that no sore nor any secondary symptoms have resulted.

When we admit that the cutaneous system is particularly prone to suffer from the venereal virus, we are sure that it does not suffer exclusively. Persons who have had venereal sores, and have not taken mercury, occasionally present, as a secondary symptom, violent pains and even decided inflammation in the deeper, as well as in the superficial portions of the fibrous system. We have seen two or three instances of periostitis of the thigh under such circumstances.

It has been supposed that syphilitic bubo is limited to one gland, and that the implication of several is evidence against the venereal character of the affection. We often find practitioners act on this idea. Yet it is certainly erroneous. Mr. Hunter, indeed, leans to the opinion, but he only leans.

"I think," he says, "there is commonly but one gland at a time that is affected by the absorption of venereal matter, which, if so, becomes in some sort a distinguishing mark between venereal buboes and other diseases of these bodies." 360.

We quite agree with Mr. Wallace upon this point.

"It is also because the venereal poison is deprived of its power of irritating the lymphatic system by passing through a lymphatic gland, that we generally find one gland only affected. We are not however to suppose, as some have here again incorrectly affirmed, that a bubo cannot be syphilitic if more than one gland be diseased; for should the absorbents which arise from the surface to which the poison had been applied, be so distributed as to pass in different directions, and consequently through separate glands, a plurality of these bodies may be affected. Indeed, I have reason to believe that this is more frequently the case than is at present conjectured; for I have sometimes found, upon the resolution of a bubo, that the swelling had been formed by the tumefaction of several glands, though before it had begun to decrease, it had all the appearance of being caused by only one enlarged gland." 345.

Syphilitic bubo has been supposed to be capable of occurring independently of any primary sore. Setting aside hypothetical arguments for the occurrence or against it, the occurrence itself would seem to be established by irresistible evidence. We have seen one case, which seemed to us to be unexceptionable. We shall introduce the sentiments of the authors before us on this point.

Mr. Hunter remarks that:—

"The first and most simple form (of absorption) is where the matter either of a gonorrhœa or chancre has only been applied to some sound surface, without having produced any local effect on the part, but has been absorbed immediately upon its application. Instances of this I have seen in men, and such are perhaps the only instances that can be depended upon; for it is uncertain in many cases whether a woman has a gonorrhœa or not. I think however I may venture to affirm that I have seen it in women, or at least there was every reason to believe that they had neither chancre nor gonorrhœa preceding, as there was no local appearance of it, nor did they communicate it to others who had connexion with them.

It must be allowed that this mode of absorption is very rare; and if we were to examine the parts very carefully, or inquire of the patients very strictly, probably a small chancre might be discovered to have been the cause, which I have more than once seen. For, when we consider how rarely it happens from a gonorrhœa, in which the mode of absorption is similar, we can hardly suppose it probable that it should here arise from simple contact, the time of the application of the venereal matter being commonly so very short. We might indeed suppose the frequency to make up for the length of time, which we can hardly allow, for the same frequency should give the chance of producing it locally. Therefore very particular attention should be paid to all the circumstances attending such cases." 357.

Mr. Wallace observes :—

"It has been, and continues to be, questioned by many persons, whether the venereal poison can enter the lymphatic system, and produce disease in it, without having previously caused some local disease upon the part to which the virus was first applied; or in other words, whether a bubo may occur without being preceded by any primary symptom.

The affirmative and the negative of this opinion are supported by different persons of equally high authority. Thus, Fallopius, Hunter, Lagneau, Swediaur, Bertrandi, &c. affirm, that bubo may be a first symptom; while A. Cooper, Delpech, and many others, support the opposite opinion.

I believe that cases of venereal bubo, which were not preceded by primary symptoms, have frequently occurred to me. I have at least treated certain cases of bubo, which were neither accompanied nor preceded by primary symptoms, but which had followed suspicious intercourse, as if they had been caused by the absorption of the venereal poison; and I have never had occasion to regret the practice. On the other hand, I have known buboes, which were not preceded by primary symptoms, to be followed by secondary symptoms when mercury had not been used in their treatment. I am, however, ready to admit, that it is difficult, if not impossible, to decide *a priori*, whether a bubo which has occurred without previous local disease be venereal or not; and also that much mischief might result from acting without great consideration in these doubtful cases.

Such buboes as are supposed to arise from the venereal poison, but which are not accompanied, or have not been preceded by primary symptoms of any kind, are often called primary buboes, and by the French *bubons d'emblée*." 346.

Dr. Colles says little.

"The history of bubo would be incomplete were we not to mention that this symptom occasionally but rarely precedes by a few days the appearance of chancre: such cases require great caution on the part of the surgeon; he should be slow to pronounce them venereal, and he should not commence a mercurial course until the chancre has appeared: this delay cannot be productive of any bad consequence, but on the contrary will encourage both patient and surgeon to enter on the mercurial course with greater confidence as to its propriety." 111.

As there are no signs by which we may distinguish a syphilitic from another bubo, independently of collateral circumstances, it is obvious that there are no means of determining whether a primary bubo, unaccompanied by any sore, is syphilitic or not. The mere circumstance of its following connexion, is certainly not enough to justify us in pronouncing the affirmative, or in subjecting the patient to a course of mercury. It appears to us, that the occurrence of secondary symptoms is requisite to dispel all doubts and to decide the necessity for specific treatment. It is possible that a combination of circumstances *might*, in a particular case, lead very fairly to the same conclusion.

Mr. Hunter remarks that he is afraid that patients have often undergone a mercurial course when there has been no occasion for it. Mr. Hunter's ideas on the means of diagnosis do not seem to us to be satisfactory.

"It will perhaps," he says, "be difficult to find out the specific difference in the diseases themselves; but I think that such buboes as arise without any visible cause are of two kinds; one similar to those arising from chancres or gonorrhœa; that is inflaming and suppurating briskly. These I have always suspected to be venereal; for although there is no proof of their being so, yet from these circumstances there is a strong presumption that they are.

The second are generally preceded and attended with slight fever, or the common symptoms of a cold, and they are generally indolent and slow in their progress. If they are more quick than ordinary, they become more diffused than the venereal, and may not be confined to one gland. When very slow they give but little sensation, but when more quick, the sensation is more acute, though not so sharp as in those that are venereal; and most commonly they do not suppurate, but often become stationary. When they do suppurate it is slowly, and often in more glands than one, the inflammation being more diffused, and commonly small in proportion to the swelling. The matter comes slowly to the skin, not attended with much pain, and the colour is different from that of the other, being more of the purple. Sometimes the suppurations are very considerable, but not painful." 365.

Prior to this, Mr. Hunter had thus laid down the characteristics of the genuine syphilitic bubo.

"The true venereal bubo, in consequence of a chancre, is most commonly confined to one gland. It keeps nearly its specific distance till suppuration has taken place, and then becomes more diffused.* It is rapid in its progress from inflammation to suppuration and ulceration. The suppuration is commonly large for the size of the gland, and but one abscess. The pain is very acute. The colour of the skin which the inflammation attacks is of a florid red."

When analysed, the distinctive characters of syphilitic bubo amount, in Mr. Hunter's mind, to singleness of gland attacked, and to acuteness of inflammation. For our own parts, we think these positions pregnant with danger, and we are sure that were they strictly acted on many a bubo would be aggravated, and many a man subjected to an unnecessary course of mercury.

The *progress* of a syphilitic bubo, or the states which it may assume, deserve consideration. The best account is given by Mr. Wallace, to whom we shall now turn.

Mr. Wallace assumes that there exists a form of bubo, of which all others are degenerations. He had previously assumed the very same thing with respect to sores. This stiff hypothesis led him into the extraordinary dilemma, of making gonorrhœa intermediate between the common venereal sore and phagedæna. It is unnecessary to remark, that these arbitrary distinctions, and these ideal standards, are simply arbitrary and ideal, and have no substantial foundation in nature. The offspring of imagination and caprice, they differ with each mind that gives them birth, and there are as many standards as observers. When we quote Mr. Wallace's history of the successive phases of bubo we quote it as a history generally correct, but not as a description of a form par excellence, of which all others are deformed productions.

"A lymphatic gland, connected by its vessels with the part to which the venereal poison had been previously applied, becomes, sometimes sooner and some-

---

* "It may be observed here, that the glands and surrounding parts being dissimilar, inflammation does not so readily become diffused as when it takes place in a common part."

times later after the application of the poison, a little enlarged, and slightly painful on handling; so as to present the characters of a small tumour, about the size of a filbert, situated under the skin, and moveable between it and the subjacent parts.

This moveable tumour, which at first seems to be caused solely by an enlargement of the gland, increases in a short time in size, and becomes more fixed, in consequence of the disease extending to the surrounding cellular tissue. At this period, the motions of the limb are somewhat painful and obstructed, owing to the morbid sensibility of the diseased gland, and to the diminished extensibility of the surrounding inflamed cellular tissue.

The tumour now soon forms a swelling of an oblong rather than of a rounded form, projecting in relief from the parts which surround it. The integuments are however, even yet, moveable on its surface, being apparently unaffected or uninfluenced, and of their natural colour.

After a time, the skin becomes red; and is then found to be adherent to the surface of the tumour, over which it could be previously moved, even after the tumour had become adherent to the subjacent and surrounding tissues. The bubo then for the most part increases with rapidity; the pain becomes of a throbbing kind; some degree of fever sets in, marked by an acceleration of pulse, an increase of heat, loss of appetite, imperfect sleep, with a general feeling of indisposition; and all these symptoms are more remarkable in the evening and during the night, than in the morning or during the day.

The swelling now becomes more prominent, and the skin more red and shining, while the tumour still feels hard and resisting; but in the course of a day or two this hardness decreases, and the swelling, which was at first somewhat doughy, soon affords a distinct sense of fluctuation. At this period the shining red skin covering the more prominent parts of the tumour begins to desquamate, so as to form scaly circles; and afterwards assumes a mottled livid appearance.

The livid patches quickly acquire a deeper colour, and often become partially black, the cuticle separates from them, and giving way, a larger or smaller quantity of thick yellowish-white matter is discharged through one or more small openings. These openings frequently present a ragged sloughy ulcerated appearance, with a red edge and cutaneous margin, and with a white pulpy substance on the inner part of the edge, as if the cavity of the bubo or the surface of the ulcer from which the pus is discharged was lined or covered by a stratum of white matter, resembling that which so frequently covers the primary sore during its stage of ulceration.

The process of destruction thus commenced in the integuments covering the tumour, extends very frequently until all that part of the skin which had been rendered very thin is removed, and until the bottom of the abscess has been so exposed, that the diseased part presents the form of an ulcer, somewhat resembling, on a large scale, the regular primary ulcer.

The process of ulceration or destruction ceases, in general, as soon as those integuments which covered the front of the tumour, and which had been very much thinned before the escape of its contents, have been removed. The ulcer thus produced is now quickly filled up by newly formed granulations, and its diameter at the same time contracting, the areola which surrounded the sore during the ulcerating stage becomes concentrated into a narrow red margin, from the inner edge of which the new cuticle proceeds; and according as the healing process advances, the outer portion of this red margin acquires a callous appearance, while the inner portion forms a red line, denoting the formation of new skin." 350.

This description is, in our opinion, a very accurate one. The different stages are portrayed with fidelity, and the changes which the gland undergoes are simply and satisfactorily explained. The duration of those stages is exceedingly indefinite. We should say, that it varies especially with the constitution and the treatment of the patient. Mr. Wallace believes, that it occupies a somewhat longer period than the regular primary ulcer. We think the course and duration of the sore much more precise than that of the bubo.

Mr. Wallace observes that the bubo may be cut short in any of its stages. He remarks, too, very justly, that there is some variety in the rapidity with which the regular bubo passes through its different stages, and in the aspect which it presents in each of them, but particularly in the fourth and fifth, or in those during which matter is formed and discharged, and the injury caused by the processes of suppuration and ulceration repaired. Thus, the integuments covering the tumour may be rendered very thin before the abscess is discharged, and the size of the opening by which it is discharged may be greater or smaller. It may in fact be so very small, that when healed, no vestige of a cicatrix can be observed; or it may be much larger, and formed by a process of sloughing rather than of ulceration; or, after the integuments covering a bubo have become remarkably thin, its cuticle may vesicate, as if a blister had been applied, and this having been followed by a kind of oozing or sweating as it were, from the surface of the excoriated skin, the bubo may shrink, and finally disappear without the formation of any ulcerated opening whatsoever;—or lastly, the process of ulceration may continue for a longer or shorter time after the contents of the abscess have been discharged, and thus form an ulcer of variable size.

It is generally supposed that in phlegmonoid abscesses, and in buboes, the process of suppuration commences in the centre of the tumour. We do not think that Mr. Wallace fairly represents the usual ideas on this subject when he states them as consisting in the belief that there is found in the centre of the bubo a small cavity, which is lined with a stratum of lymph; that purulent matter is secreted into this cavity by its lining membrane; and that the germ of the future abscess being thus formed, its extent is afterwards gradually enlarged, by a process which Mr. Hunter has called progressive absorption. We believe on the contrary that the notions of the best informed pathologists are more accurately expressed, by the description we are about to quote, and which Mr. Wallace advances as original.

"If a phlegmonoid tumour be examined at its commencement, it will be found that its vessels contain a preternatural accumulation of blood; that there is a preternatural accumulation of lymph or serum into the interstices of the texture composing it; and that this texture is rendered much more soft and more fragile, and hence more easily torn, than in its natural state. It will also be found, that in proportion as the disease advances, the tumour becomes liquified in the centre, where the morbid action is more violent, than at the circumference; that if the swelling be then incised, the surface of the section will present an irregular cavity, filled with matter more or less fluid; that on the outside of this cavity the texture of the tumour is soft, or somewhat like a sponge, mixed with purulent matter, and in proportion as we recede to the circumference, the diseased tissue seems more firm and more vascular, and contains less of this matter.—Finally, the whole tumour becomes in time perfectly soft or liquid throughout, and the original texture, thus softened, is mixed with the fluids previously secreted into the interstices of the diseased tissue." 353.

*Properties of the Discharge from Bubo.* It is a question of some importance, whether the matter secreted by a syphilitic bubo is capable of conveying the syphilitic poison to other persons, or to other parts. In reference to this question, we may observe, in the first place, that, in some instances, the ulcers produced by suppurating buboes resemble very closely primary syphilitic ulcers. In the majority of cases, we should say, from what we have seen, that they do not wear this resemblance. Their presenting it is the exception, not the rule. Nor, in the majority of cases, does the discharge from the bubo contaminate contiguous parts, although, were it charged with the venereal virus, it would most probably do so.

Mr. Wallace informs us, that he has made many direct and positive experiments on this point. "I have," says he, "perhaps some hundred times, when opening buboes, inoculated with the matter discharged the individuals themselves; and, except on three occasions, I never succeeded in producing any specific effect; but on these three occasions, there resulted ulcers which presented throughout their whole course the characters of the primary syphilitic ulcer. From these experiments we are authorized to conclude, in the first place, that the matter of bubo is capable of producing primary syphilis; and in the second place, that the inoculation of this matter very often fails to cause a specific disease."

The matter must be allowed to rest thus undetermined for the present. A difficulty attends the practice of inoculation with syphilitic matter. Persons not already infected cannot be got in any numbers to submit to it. Persons uninfected *may* have their susceptibility to fresh infection modified, so as to vitiate the results. Our knowledge of the laws of the contagion of syphilis is, after all, extremely imperfect.

*Treatment of Bubo.* All previous enquiries must centre, at length, in this. The treatment of bubo is by no means simple in all cases, and probably there are few surgeons possessed of such discrimination as not to commit some practical blunders occasionally.

Bubo, for the purposes of treatment, may conveniently be divided into three stages;—the first, that in which the bubo constitutes a small moveable tumor beneath the skin, rather tender when disturbed, but little (it must be slightly) inflamed, and unaccompanied by inflammation of the surrounding cellular membrane or skin; the second stage, that of augmented volume and decided inflammation of the bubo, with implication of the cellular membrane and skin; the third stage, that of suppuration; the fourth, that in which the bubo has become an ulcer.

In the two first stages, the object of the surgeon will usually be to effect resolution. In the third, he is anxious to open the abscess in the most favourable manner. In the fourth, he wishes to heal the ulcer. Throughout all the stages he is alive to the necessity for removing, by the specific influence of mercury, the specific disease from which the bubo has resulted, and he is happy when he finds that this general indication is not thwarted by the state of the local affection. Unfortunately it, not seldom, is so.

*Resolution.* In the treatment of syphilis, as of gonorrhœa, it is always requisite to bear in mind, that the specific malady may be mixed up with more or less of inflammation. A certain quantum of inflammatory action, is of course, an integral part of the complaint, which appears, in fact, to be essentially a modification of that action. But, if it passes a certain amount, the inflammation becomes a disease per se, and requires to be moderated by the means which are usually adapted for its moderation. It is impossible, we think, for any one to treat either syphilis or gonorrhœa successfully, who does not make this the basis of his remedial views.

If this is true of a syphilitic ulcer, it seems, à fortiori, true of a syphilitic bubo. The latter is essentially a more inflammatory affection than the former, a less direct and specific effect of the poison.

If a bubo is swelling rapidly, extremely painful, and implicating extensively and quickly the surrounding cellular membrane, that is in our opinion, sufficient evidence of excess of inflammation, and of the necessity of adopting antiphlogistic measures.

In such a case, we should advise, and we resort to the application of leeches,*—evaporating lotions, or bread poultices made with Goulard water—salines—aperients—rest. After a sufficient application of leeches we have seen much benefit from the use of blisters. They may be put upon the bubo. In many instances, we have seen impending suppuration *apparently* prevented by them.

There are no points in the practice, nor any in the theory of medicine, on which there are not wide differences of opinion. If one man recommends a poultice, another is pretty sure to denounce it. It cannot, then, excite surprise to find that some persons are averse to antiphlogistic measures in the treatment of syphilitic bubo. They contend that leeches are highly injurious. They do not affect to explain this as a matter of reason, or on the grounds of analogy, but as the simple result of experience. From this there appears to be no appeal, and all that can be done is for each man to record his experience, and to leave the issue to the accumulated observations of many. For our own parts, we must observe, that so far as we can draw correct conclusions from the facts that we have witnessed, they *seem* to be decidedly in favour of leeches, when syphilitic bubo is attended with decided inflammation. We are the more disposed to lean upon our observations, as they are fortified by reason and analogy.

The superiority of warm or of cold applications has been much litigated, and is still undetermined. It appears to us, that, as a general question, it is insusceptible of a decided affirmative or negative either way. In the greater number of cases cold applications have seemed to us to be most serviceable. But it has occasionally happened that the change from cold to warm applications has been productive of evident service, and different individuals seem to have peculiarities in relation to the benefit they derive from such measures.

We now turn to our authors.

Mr. Hunter distinctly lays down the principle on which we have insisted. We shall shew, presently, that his remedial precepts are to be

---

* Leeches, in these as in most cases, should be applied in the immediate neighbourhood of the swelling, rather than upon it. The object to be gained, is the drawing blood *from* the part, not *to* it. Whoever has watched the effects of leeches, must have remarked the tendency to congestion in the vessels of the part on which they have been used.

received with some degree of reservation. On this point, however, they seem unexceptionable.

"There are many cases in which mercury by itself cannot cure. Mercury can only cure the specific disposition of the inflammation; and we know that this disease is often attended with other kinds of inflammation besides the venereal.

Sometimes the common inflammation is carried to a great height; at other times the inflammation is erysipelatous, and, as I suspect, often scrofulous. We must therefore have recourse to other methods.

Where the inflammation rises very high, bleeding, purging, and fomenting are generally recommended. These will certainly lessen the active power of the vessels, and render the inflammation more languid; but they can never lessen the specific effects of this poison, which were the first cause, and are still in some degree the support of the inflammation. Their effects are only secondary; and if they reduce the inflammation within the bounds of the specific, it is all the service they can perform. If the inflammation be of the erysipelatous kind, perhaps bark is the best medicine that can be given; or if it be suspected to be scrofulous, hemlock, and poultices made with sea-water, may be of service."

Making allowance for the indifferent *medical* knowledge displayed by Mr. Hunter, in considering bark as a specific for erysipelas and hemlock as one for scrofula, his general ideas are much to be praised.

In the work of Dr. Colles, the last that has been published on the subject, it appears to us that the spirit of discrimination on this point has rather retrograded than advanced. Dr. Colles does not endeavour to separate the accidental from the necessary conditions of bubo, or, rather, he does so in a very indistinct and unimpressive manner. We shall quote the brief observations of this author, that we may not, unintentionally, do him an injustice. In quoting them, however, we must state that we differ from him on two points—first, on the freedom with which he gives mercury during an inflammatory condition of bubo—secondly, on the small account he makes of local depletion and of antiphlogistic treatment generally during this same condition. With the expression of this dissent, we quote the passage in question.

"When chancre and incipient bubo co-exist, it is admitted, that the treatment by mercury should be precisely the same as if the chancre alone were present; and accordingly we find, that as the latter improves, so the swelling and tenderness in the former subside: even should the bubo, in consequence of neglect have acquired great size, and have advanced so far toward suppuration, that the integuments shall have become red, still the treatment should be the same as for the chancre alone. When the ptyalism is commencing, the inflammatory condition of the bubo will for a day appear rather aggravated, but when the ptyalism is established, then the inflammation obviously begins to subside. Some advise the use of mercury to be postponed until the gland has suppurated, and the inflammation has been reduced by letting out the matter; I do not think this delay is necessary, though I admit we should first adopt antiphlogistic means to subdue or mitigate the febrile symptoms which have been excited by the inflamed condition of the bubo; this being accomplished, there is no reason why we should longer defer the use of mercury. But should we meet a case, in which, while the chancre is improving, (ptyalism being established,) the bubo becomes larger, and is attended with an increase of inflammatory redness, and a painful sensation like that of scalding, we are then to infer that the mercury is disagreeing; we should, under such circumstances, discontinue it for a few days, direct brisk purgatives, and the most strict quietness, also the horizontal posture.

The amendment in the bubo will assure us that such a line of practice was most judicious, and we may then resume the mercury as soon as we observe its effects on the system beginning to decline, using it perhaps in less powerful doses." 99.

Mr. Wallace admits the necessity for antiphlogistic treatment in the inflammatory state of bubo. We think *he* leans too much on mercury, and drives a sort of bargain between principle and empiricism. He observes that, when the bubo is of some size, adherent to the skin, which is more or less extensively inflamed, and accompanied with throbbing pain and fever, it is indispensable to bring the system with the greatest possible rapidity under mercurial influence ; and also to combine with the use of mercury such remedies, both local and constitutional, as are most likely to subdue or control inflammatory action. Among these may be mentioned local, and in full habits perhaps general blood-letting,—large doses of tartrate of antimony,—the application of evaporating and saturnine lotions, together with abstinence and perfect quietness.

Mr. Wallace, therefore, combines decided antiphlogistic treatment and impregnation of the system with mercury "with the greatest possible rapidity." To this method of proceeding we object most strongly. It is the old and the pernicious method which has ruined many constitutions, and, if persevered in, will surely ruin many more. Persons of a vigorous frame may resist its destructive influence, nay will here and there be benefited by it ; but the majority of individuals, especially in towns, are quite unable to bear up against it, and will most certainly in the gross, and in the long run, be seriously damaged by any such measures.

It must be remembered, that inflammation of the lymphatic glands is inflammation of a tissue into which cellular membrane enters largely. Such inflammation is not so likely to be put down by active antiphlogistic treatment *and* mercury, as inflammation of the serous or the fibrous membranes. This is certainly the case with inflammation of the general cellular tissue, and we think it the case with syphilitic inflammation of the glands. But whether the latter be true or not, we are sure that the combination of mercury in large doses and decided antiphlogistic treatment, is what some *constitutions* cannot bear, and what many have sunk under. With persons of irritable habits, buboes or any local affections are apt, under such treatment, to run to mischief, and suppuration, the thing dreaded, is precipitated by the over-debilitating measures employed for its prevention. Whether our readers agree with us or not in these opinions, we would advise them, at all events, not to adopt such measures as those prescribed by Mr. Wallace, without keeping a very careful eye upon their patients, and being ready to suspend their powerful remedies if any inopportune symptoms should arise.

Supposing that the inflammation of a bubo is trivial, or that it has been rendered so by treatment, and that the time for administering mercury has arrived, we naturally inquire what method of doing so is best.

It is impossible, here, to go into the answer in detail. In the last Number of this Journal, we devoted an article to the subject of the administration of mercury, and dwelt on what appeared to be the best and the worst modes of regulating it. It would be useless re-opening the question, and all that appears necessary is to consider one or two points which are concerned in the special management of bubo.

A. Mr. Hunter was of opinion that the resolution of inflamed bubo not only depends principally upon mercury, but "almost absolutely upon the quantity that can be made to pass through it." Nay, he went so far as to assert, that if the bubo had come to suppuration "the cure depends upon the same circumstances."

This is only a more extensive form of the proposition that we have already dwelt on when alluding to the opinions of Mr. Wallace. It is true, that, if our notions of the specific influence of mercury on syphilis are accurate, the quantity of the specific received into the system, is an important element in effecting the cure. To a certain extent, the quantity of mercury employed must also form a necessary element in the cure of syphilitic bubo. But it is not so certain that to *prevent suppuration* in that bubo, a large amount of mercury is requisite, and this is a material point.

Mr. Hunter's opinions with respect to the quantity required are expressed in a tolerably decisive manner. He remarks that it must be proportioned to the obstinacy of the bubo, but care must be taken to stop short of certain effects upon the constitution. If it be in the first situation, and yields readily to the use of half a drachm of mercurial ointment, made of equal parts of quick-silver and hog's lard, every night, and the mouth does not become sore, or at most only tender, then it will be sufficient to pursue this course till the gland is reduced to its natural size; and this probably will be a good security for the constitution, provided that the chancre, which may have been the cause of the bubo, heals at the same time. If the mouth is not affected in six or eight days, and the gland does not readily resolve, then two scruples or a drachm may be applied every night; and if there be no amendment, then more must be rubbed in; in short, if the reduction is obstinate the mercury must be pushed as far as can be done without a salivation.

If there be a bubo on each side, then there cannot be so much mercury applied locally to each; for the constitution most probably could not bear double the quantity which is necessary for the resolution of one. But in such cases we must not so much attend to the soreness of the mouth as when there is but one; however, we must allow the buboes to go on to suppuration, rather than affect the constitution too much by the quantity of mercury; and therefore when there are two buboes they are more likely to suppurate than where there is only one.

It must be owned that these opinions are exposed to some objection. Thus, the reduction of the bubo and the *healing* of the chancre, are considered by Mr. Hunter to demonstrate the propriety of discontinuing the use of mercury. Yet there are many cases where mercury ought to be still exhibited, and the state of the cicatrix of the sore, as well as the time during which the mercury has been employed, are the principal circumstances which should influence our decision.

B. As bubo results from extension of the syphilitic virus, and of inflammatory action, along the absorbents to their glands, it has been naturally supposed, that the specific remedy should travel by the same route. Mr. Hunter carried out this idea to its full extent.

"Mercury," said he "is to be applied in the most advantageous manner; that is, to those surfaces by an absorption from which it may pass through the diseased gland; for the disease there being destroyed, the constitution has less chance of being contaminated. The powers of mercury may often be increased from the manner in which it is applied. In the cure of buboes it should always be made to pass into the constitution by the same way through which the habit received the poison; and therefore, to effect this, it must be applied to the mouths of those lymphatics which pass through the diseased part, and which will always be placed on a surface beyond the disease.

But the situation of many buboes is such as not to have much surface beyond them, and thereby not to allow of a sufficient quantity of mercury being taken in in this way; as, for instance, those buboes on the body of the penis arising from chancres on the glans or prepuce.

These two surfaces are not sufficient to take in the necessary quantity to cure those buboes in its passage through them; therefore whenever the first symptoms of a bubo appear, its situation is well to be considered, with a view to determine if there be a sufficient surface to effect a cure, without our having recourse to other means. It is first to be observed, whether the absorbent vessels on the body of the penis are affected, or the glands in the groin. If the disease be in the groin, it must be observed in which of the three situations of the bubo, before taken notice of, it is; whether on the upper part of the thigh and groin, and on the lower part of the belly before Poupart's ligament, or near to the pubes. If they are on the body of the penis, this shows that the absorbents leading directly from the surface of absorption are themselves diseased. If in the groin, on the upper part of the thigh, or perhaps a little lower down than what is commonly called the groin, then we may suppose it is in the glands common to the penis and thigh. If high up, or on the lower part of the belly, before Poupart's ligament, then it is to be supposed that those absorbents that arise from about the groin, lower part of the belly, and pubes, pass through the bubo; and if far forwards, then it is most probable that only the absorbents of the penis and skin about the pubes pass that way. The knowledge of these situations is very necessary for the application of mercury for the cure by resolution, and for the cure after suppuration has taken place.

The propriety of this practice must appear at once when we consider that the medicine cannot pass to the common circulation without going through the diseased parts; and it must promote the cure in its passage through them; while at the same time it prevents the matter which has already passed, and is still continuing to pass, into the constitution from acting there, so that the bubo is cured and the constitution preserved." 369.

It is probably correct to believe that mercurial inunction is the most efficient mode of administering mercury. Yet probably also, more importance has been attached to this direct method of exhibiting it than it deserves, for we find that buboes, as well as other venereal symptoms, are cured satisfactorily enough by the blue pill. If mercurial inunction is on the whole the best, it is not merely from its travelling the same route as the poison, and, if we may say so, overtaking it, but probably from its producing less disturbance in the system, than mercury received by the stomach occasions. We find that mercurial inunction is as well adapted for secondary symptoms as for primary, although, in the former case, the rationale of its action is not so direct.

Mr. Hunter, indeed, appears to be extravagantly given up to the idea that the medicine ought to travel post after the poison. He does not so much as mention the administration of mercury internally for bubo, and he points out the exact spots for rubbing in the ointment, according as the bubo varies in its situation. If the bubo be in the groin, then he tells us that it is necessary to rub the mercurial ointment upon the thigh. This surface will in general absorb as much mercury as will be sufficient to resolve the bubo, and to preserve the constitution from being contaminated by the poison that may get into it; but if resolution does not readily take place, then we may increase the surface of friction, by rubbing the ointment upon the leg.

But if the bubo be on the lower part of the belly, that is, in the second situation, then the ointment should be rubbed also upon the penis, scrotum, and belly; and the same if the bubo should be still forwards; for probably those glands receive the lymphatics from all the surfaces mentioned, as well as from the thigh and leg.

When buboes occur, in women, between the labia and thigh, the mercury, Mr. Hunter informs us, may be rubbed in all about the anus and buttock, as all the absorbents of those parts probably pass that way; we know at least that they do not pass into the pelvis by the anus, but go by the groin. Other means of introducing mercury must be recurred to, as is recommended in the case of men; but still it will be proper to rub in on these surfaces as much as possible. These statements prove how much importance Mr. Hunter attached to the site and practice of mercurial inunction, and, perhaps, that he overrated the necessity for paying extreme attention to either.

We have already observed, that, so far as we have been enabled to judge, inunction is the best mode of exhibiting mercury. Yet it is little practised. The objections to it are its inconveniences. Syphilis is a disease which, in most instances, is concealed. Inunction is not only a troublesome, but it is a tell-tale sort of remedy. Private patients generally dislike it.

On the whole, the treatment of syphilitic bubo by mercury may be loosely summed up in the following conclusions: *that,* when the presence of excessive inflammation does not, for the time, forbid it, mercury should be freely, but cautiously, employed; *that,* the quantity of mercury administered should be regulated by the consideration of all the circumstances of the case, and not merely by a reference to the individual symptom—bubo; *that,* the termination of the course should not hinge on the removal of the bubo only; *that,* on all these points, the surgeon must be guided by his general knowledge of syphilis, and by the general principles of its treatment; *that,* mercurial inunction is, cæteris paribus, the best mode of using mercury for bubo, but not at all indispensable.

*Treatment of Bubo in the Stage of Suppuration.*—We have failed in the attempt, and this should not often happen, to prevent the suppuration of a bubo. Its management in this stage now devolves upon us. Conflicting opinions have been expressed and acted on, and the reader of different works feels a difficulty in determining what plan to adopt.

A. Occasionally, a bubo has all the appearances of having actually suppurated, yet either those appearances are false, or the matter formed may be absorbed. Mr. Hunter mentions a case of this kind, in which sea-sickness cured the bubo; he mentions it for the purpose of introducing emetics to our notice.

"Vomits have been of service in resolving buboes, even after matter has been formed in them, and after they have been nearly ready to burst; this acts upon the principle of one irritation destroying another; and sickness and the act of vomiting perhaps give a disposition for absorption. A remarkable instance of this kind happened in an officer who had a bubo at Lisbon. It came to fair suppuration, and was almost ready to burst. The skin was thin and inflamed, and a plain fluctuation felt. I intended to open it, but as he was going on board a ship for England on the day following, I thought it better to defer it. When he went on board he set sail immediately, and the wind blew so very hard that nothing could be done for some days, all which time he was very sick, and vomited a good deal; when the sickness went off he found the bubo had disappeared, and it never afterwards appeared. When he came to England he went through a regular course of mercury." 370.

B. After other means have failed, and suppuration seems inevitable, we have seen repeated blisters effect its gradual dispersion. We should say, indeed, that if other methods seem likely to be unsuccessful, the patient should always be offered the chances which blisters give. They are painful remedies—they frequently prove useless—but now and then they decidedly seem to prevent suppuration.

Dr. Colles recommends blisters in a particular condition of bubo. We are not disposed to limit their use to that condition, but we admit their peculiar adaptation to it.

" We sometimes meet with the following condition of a bubo,—a large, indurated, indolent mass, the integuments but little or not at all discoloured, with sometimes a slight tendency to inflammation and suppuration, and yet the patient has used mercury so as to affect his system; in such a case I think blisters will be found most efficacious; they should be applied repeatedly but not retained longer on the part than five or six hours. Some advise us, if we feel a softened spot in the centre, or in any part of this tumour, to apply the kali purum, with the hope not only of letting out the matter, but also of exciting a healthy inflammation in the part; this practice, however, entails the additional inconvenience of an open ulcer without accelerating the dispersion of the bubo as efficaciously as blisters do. Many cases of this sort are ascribable to an injudicious use of mercury, and therefore we must at the same time apply ourselves to restore the system to a more healthy state." 103.

C. We have occasionally seen a bubo advance apparently towards suppuration, unchecked by any remedies employed. It has seemed to be on the point of breaking, the skin being extensively reddened and thin, and the swelling presenting, if not actual fluctuation, at all events a boggy state, which closely counterfeits it. If the surgeon plunges a lancet into the swelling, bloody serum, and not pus, issues, or, perhaps a thin sero-purulent matter may flow. Such buboes, however unpromising they may appear, not unfrequently do very well. The cuticle desquamates, the swelling gradually subsides, and resolution is effected after all. We have seen this occur under poultices, blisters, and such various modes of general treatment, that it is difficult to say what particular method best suits this condition of bubo.*

---

* Mr. Wallace appears to allude to the same fact as we have done. Having remarked that, even when suppuration has occurred, the pus is sometimes absorbed, he proceeds :—" But, on the other hand, should the process of suppuration have advanced more slowly, having been long in commencing; should the bubo be attended by comparatively little pain and heat, or inflammation; should the skin covering it be somewhat flaccid or wrinkled, with a strong propensity

D. If suppuration is unequivocal, the skin growing thinner, and absorption of the matter out of the question, the surgeon must determine whether he will let the bubo burst, or rather let the skin be more or less destroyed by ulceration—or, whether he will open the abscess. He must first, however, determine another thing—whether he will continue mercury, supposing that he has used it up to this point. For our own parts, we must say, that, when suppuration seems inevitable, we think it best to discontinue the administration of mercury, and not to resume it until the stage of granulation has commenced. We treat the abscess as a thing per se, an accidental consequence of an accidental amount of inflammation, and not as a part of the special disease. Without entering on reasons which must be more or less hypothetical, and which may be true or may be false, we are convinced, as a matter of simple observation, that if suppuration is decidedly advancing, it is better to withhold the mercury. The continuance of its use seems to us to tend to make the suppuration more extensive, and the subsequent stage of ulceration more severe.

Mr. Hunter alludes in a specific manner to this subject. He adopts, it will be seen, a middle course; and, considering that, at the time when Mr. Hunter wrote, mercury was thought to be more indispensable than it now is, his moderate opinions tell in favour of the plan of suspending the administration of mercury altogether.

"It may admit," observes Mr. Hunter, "of dispute whether the application of mercury should be continued or not through the whole suppuration. I should be inclined to continue it, but in a smaller quantity: for although the parts cannot set about a cure till opened, yet I do imagine that they may be better disposed to it; and I think that I have seen cases where suppuration has taken place, although under the above-mentioned practice, that were very large in their inflammation, but very small in their suppuration, which I imputed to the patient's having taken mercury in the before-mentioned way, both before and while suppuration was going on." 374.

Mr. Wallace speaks boldly to the point.

"If an incision be made into a bubo while the system is impregnated with mercury, or if mercury be exhibited soon after an opening has been made, or soon after a bubo has spontaneously opened, we shall be in danger of exciting a troublesome form of disease, which it may be afterwards very difficult to control. Therefore, when we are of opinion that a bubo is not likely to be resolved by mercury, and that it must be allowed to open spontaneously, or be opened by art, we are to desist from the use of this medicine until suppuration is completed and the matter discharged; or until an incision has been made into the tumour, and that, by antiphlogistic and emollient treatment, &c. the wounded part has been brought into a state of tranquillity. We are to be equally careful of opening a bubo when the system is already impregnated with mercury." 364.

It has generally appeared to us, that, when the matter was coming forward, salines and aperients have answered best.

---

to desquamate; and should the habit of the patient be less full or plethoric; I do not despair of causing the resolution of the bubo and the absorption of its contents, particularly if mercury has not been previously used."

The only difference between Mr. Wallace and ourselves is this—that he limits the power of effecting resolution to mercury—while we believe that several modes of treatment may possess it.

E. We are not aware of any good reason for allowing the bubo to be opened by the natural process of ulceration of the skin. But the common sense and experience of surgeons have ever supplied reasons enough to the contrary.

F. The superior advantages of opening the abscess by incision or by caustic have divided in some measure the opinions of surgeons. It appears to us, that, as a general rule, there are no valid reasons for preferring caustic, nor for opening a bubo in a different manner from that in which we open other abscesses. The destruction of the skin by caustic is painful, clumsy, slow, and must necessarily destroy more or less integument. In irritable habits, too, it may possibly induce such destructive action as is not very easily put down. The following case, though not quite in point, is sufficiently so to lead us to publish it. It gave us a warning, and may give the same to others.

*Case.* In 1833, a young man, an assistant in a soda-water manufactory, came under our care. He had had a suppurating bubo in the right groin. This had been opened, the cavity was large, and there was a quantity of thin blueish skin flapping over it, and apparently interfering with the processes of reparation. It occurred to us that it would be better to destroy this integument by caustic. We did so with the potassa fusa. In a day or two the bottom of the cavity and the surrounding skin assumed an angry appearance, a greyish sloughy surface having formed upon the former, a red blush hanging over the latter. This angry appearance increased—a large phagedænic sore resulted. The cavity deepened by ulceration and by sloughing, and extended in circumference by the same processes. At the end of seven or eight days it was of the size of a small saucer, and the femoral artery, covered with an ashy, spongy, sloughy layer of granulations, was seen beating at the bottom of the sore. Hæmorrhage was expected every hour, and assistants were stationed with the view of controlling it by immediate pressure. We had just seen the young man one evening, and were leaving the room, when we heard the cry of the vessel having given way. We rushed to his bed-side, and saw a jet of arterial blood issuing from the ulcer. In an instant, nearly a pint of blood had been lost. We thrust our thumbs into the sore at the bleeding spot, and, fortunately, the pressure stopped the hæmorrhage. We kept the thumbs in the wound for a couple of hours, until, in fact, we had lost all sensation in them. Then, having directed compression on the artery above the sore and below it, we cautiously substituted lint and a pressè-artere for the hand, and, by means of assistants, kept them applied without change of position for twenty-four hours or more. In the mean time we had given the patient large quantities of port-wine and opium, indeed had stupefied him with them. Our delight may be conceived at observing, at the end of the twenty-four hours, a healthier appearance in the ulcer, and no immediate disposition to hæmorrhage. We plied the stimuli, dressed the wound, granulations came, and ultimately the patient recovered perfectly—an event that, at one time, we did not venture to hope for. As we remarked before, this case read us a lesson. Let those who will, apply caustic freely to the groin; we confess that we are rather afraid of it.

The main argument urged for the application of caustic, is the inability of the skin to recover itself. But there seems no reason, à priori, why the skin should ulcerate or slough in bubo more than in other glandular abscesses. If, in point of fact, it does, the surgeon is led to investigate the cause, and to inquire whether *he* is not that cause. In our conscience, we believe that he generally is.

The continued and excessive use of mercury, conjoined with those antiphlogistic remedies which the previous inflammation has naturally led to, must tend to lower the powers of the system and to increase the suppurative and ulcerative processes. In proportion as those means have been abused, will the destructive predominate over the reparative actions. If, under such circumstances, the skin is allowed to become very thin, before an opening is made in it, we cannot be surprised at the vital powers of the integument, dissected as it is from the subjacent parts, being unequal to its preservation, or to the effort of producing granulations.

If these views are correct, it follows that if mercury is not pushed too far, if antiphlogistic measures are not pursued too long, and if the integument is not suffered to become too thin, its destruction is not necessary nor even probable. Nor do we believe that it is. We can only say that we do not find the skin ulcerate or slough, nor do we meet with cases in which caustic is required to remove, by a quick process, integuments which must otherwise be removed by a slow one.*

G. If incision is preferred to caustic, two or three questions are still open.

Is an early opening or a late one preferable ?

Is a small incision or a free one advisable ?

On these points we find no explicit information in the pages of Mr. Hunter.

The opinions of Dr. Colles are expressed in few words.

"The opening of the bubo should be delayed until the tumor has pointed, and the skin has become very thin; an earlier opening will induce an increase of pain and fresh inflammation, which will not subside in less than three or four days: by delay, also, there is a chance that the matter may be absorbed, and the patient be thus saved from all the annoyance attendant on an ulcer in the groin.

In most cases I advise the opening to be made in the thinnest part of the integuments, and only large enough to admit of the easy escape of the matter.

There is one particular form of bubo, in which a very different rule for opening it must be adopted; I allude to that which has acquired a large size, in which the tumor does not point or become conical, but assumes a flat surface, the whole of the integument becoming very thin and discoloured; in such a case we should cut through the entire length of this thin covering of the abscess. Were we only to make a small opening, we should find that the discharge would

---

* In reference to the pain produced by incision and by caustic, we may quote a fact mentioned by Mr. Hunter:—"I once opened two buboes in the same person, one immediately after the other. The first was with the lapis infernalis, which gave him considerable pain, and therefore he would have the other opened with a lancet, as the pain would only be momentary. But it was great, and the soreness continued long, while there was no pain in the other, deadened by the caustic, after it had done its business."

We doubt whether this is generally the case. The pain of a cut does not usually last long. The pain occasioned by caustic is not usually evanescent.

continue very long, that it would be thin and of bad quality, and that at the end of five or six weeks there would be no prospect of healing; for the integuments will have lost so much of their vitality, that they will either be removed by ulcerative absorption, or will have curled and turned in, and then the surgeon will be compelled to apply the caustic, or the knife, for their removal." 101.

Mr. Wallace is seldom indisposed to refine, and occasionally, too, he uses words so liberally that the reader feels some difficulty in determining the sense. But this may be collected from his diffuse remarks:—first, that, the incision should be proportioned both in its depth and extent to the size of the tumor; for, unless it be made deep, we may not reach the purulent focus; and unless it be made extensive, or through such parts as are in progress to suppuration, we shall not stop this process; and, before it be completed, the opening we have made may close up from tumefaction, and the patient be thus exposed to the necessity of a fresh operation, or else to await the discharge of the matter by the natural actions of the part. Whereas, if we make an incision sufficiently extensive, we shall not only avoid these evils, but also diminish very considerably the extent of the disease. In fact, an incision into a bubo, when in the state of incipient suppuration, will in general as effectually put a stop to its progress as it will to that of an anthrax when in an analogous state;—secondly, *that*, if the incision be deferred until the process of suppuration be complete, a small puncture will be sufficient; for, as soon as the containing matter is discharged, the cavity of the abscess will contract, and its sides become gradually agglutinated by the cohesion of the lymph with which they are lined.

"It still, however," continues Mr. Wallace, "remains to be determined, whether we should prefer opening a bubo in its earlier stage of suppuration, with the object of stopping its further progress—as we do in cases of anthrax—or wait until the suppurative stage is more fully advanced. This is a question, however, which the circumstances of each case must decide for itself. I shall only observe that an early incision into a bubo will in general, as already said, stop its further progress; but it will of course give more pain than a simple puncture at a more advanced stage of the disease. I would therefore leave the choice to the patient; and we shall find that some will prefer an early incision, with the view of cutting short the disease; while others will prefer waiting until the disease be *ripe*—to use a popular form of expression." 363.

These statements will we think appear startling to most surgeons of experience. An incision into a carbuncle cuts it short, for these reasons. Carbuncle is inflammation and sloughing of the subcutaneous cellular membrane. If no incision is made, pus and lymph are deposited in the cells of the tissue, and not only interrupt the vascular supply to them, but, subsequently that to the skin itself. The parietes of the cells consequently die, and the skin dies over them afterwards. An incision, made sufficiently early, discharges the cells of the pus and lymph, relieves the tension of their parietes, and prevents more or less the interruption of the vascular supply to them and to the skin. It is very dubious whether the case of suppurating bubo is analogous in principle or in detail to that of anthrax—it is still more dubious whether an early incision into it will cut it short. Mr. Wallace's argument is extremely unsatisfactory;—the plan of early incision, says he, is most successful, but it is painful, and patients will not submit to it. If it were merely painful, we are sure that most surgeons would recommend, and that many patients would be found quite willing to submit to it. But we feel confident that not only is it very painful, but it would not always cut the bubo short. If an opening is made too early, into a phlegmonous abscess, and especially into an abscess in a gland, one or two focuses of suppuration may be evacuated, but others subsequently form, and other openings are required for them. This, we have little doubt, would happen in many instances with bubo; nay, when the opening has been made early, we have ourselves seen it happen. Another argument against precipitate incisions is the probability that in irritable constitutions they might excite phagedænic action. Any one that has seen much of syphilis will always have the fear of this before his eyes, when he pokes his lancet or his probe into inflamed venereal swellings. A third, and not a trifling consideration militating against Mr. Wallace's advice, is the certainty, that, were all buboes so incised at an early period, the patient would often undergo much pain and some degree of risk for nothing, as many buboes which bid fair to suppurate, do not do so after all.

So far as we can determine the value of the cases we have seen, and draw correct conclusions from those data, the following appears to us to be the best mode of proceeding.

1. To wait until the abscess has fairly pointed—that is, until a considerable central or other portion of the tumor has softened, and the integument has become thin in some definite part. The amount of softening and of thinning is insusceptible of verbal description. It is *not* wise, we conceive, to wait until the integument has grown *very* thin, to any material extent, nor to allow it to assume a blueish colour. If these conditions are permitted to occur, it is not improbable, that, after incision, the skin will ulcerate or slough.

2. The opening should be as dependent as possible. Its direction should neither be in a horizontal nor in a vertical plane. If, in the former, each motion of the thigh will act on the lips of the wound, separating or approximating them, and by this disturbance interfering with the subsequent processes of reparation; if the opening is vertical, it is not exposed to this inconvenience, but it does not sufficiently evacuate the abscess. An oblique incision combines, in some measure, the advantages, and avoids the inconveniences of both the others.

3. We have not found, in buboes of any magnitude, a small opening answer. The matter has collected below the wound, and a sinus has, not unfrequently, resulted.

We have tried two methods of obviating this annoyance. One is the employment of a seton. When the bubo is in a condition to be opened, we have made a small incision into the most prominent point, as nearly as possible one third from the highest point of the circumference of the bubo. Into this opening we have introduced an eyed probe, armed with a thin seton of silk, or lint. Directing the probe to the most dependent part of the abscess, we have made the skin project upon its point, and cut down upon the latter. The seton-thread is then drawn from opening to opening, and retained until the cavity is pretty well filled by granulation. We tried this plan extensively in the Lock Hospital, and it answered there extremely well. Of course, it is only necessary in cases of bubo of some size.

A second mode of treating such buboes is by laying them open in the inferior two-thirds of their extent. An opening is made, as in the application of the seton, about one-third from the upper margin of the abscess. Into this opening a director is passed, and its point made to project under the skin at the most dependent recess of the cavity. A sharp-pointed bistoury is run along the director, pushed through the skin at the dependent point alluded to, and the intermediate skin between the superior opening and that point divided. This plan answers extremely well. If properly executed, we might almost say that a second cut is *never* requisite. At all events it will very rarely be so. The surgeon should take care to observe the rule we have given with respect to the direction of the cut—as well, at least, as he can.

We are quite sure that small openings for large buboes are trifling with the time and hopes of the patient. Sinuses form, fresh openings are necessary, unnecessary pain and disappointment are inflicted, and the results are any thing but satisfactory either to surgeon or to patient. We have seen a patient confined to his bed by a succession of those sinuses for upwards of twelve months—we have seen many patients so confined for several months.

H. The opening of the abscess may be said to be the signal for tonics. We do not say that they are to be given with the eyes shut, but we do say that the principle then is to support the patient. On the means of doing so we shall not dwell. Quinine, sarsaparilla, good diet, a certain quantity of the stimulus that the individual has been accustomed to take, will naturally occur to the mind of the surgeon. Support, rest, poultices, are the rule—any thing else the exception. Granulations are the things desired. Those means contribute to their coming. Mercury does not, and mercury, as the rule, should be avoided. On this point we differ from Mr. Hunter. He observes that the bubo, after it is opened, is to be dressed "according to the nature of the disease, which, I have already observed, is often so complicated as to baffle all our skill. The constitution at the same time is to be attacked with mercury, either by applying it internally or externally: if externally, it should be applied to that side and beyond where the bubo is, as I before directed in treating of the resolution of buboes, for it may have some influence on the disease in its passing through the part."

We feel pretty sure that the bubo baffled the skill of Mr. Hunter, the more frequently on account of his ideas respecting mercury.

Mr. Wallace's injunctions seem to us much more safe.

> "As a general rule, mercury is not to be employed in such cases until the stage of granulation has commenced,—and for the same reason as we refrain from its employment in the ulcerative stage of the primary ulcer. In fact, if it be used at this period, we run some risk of exciting an increase of the ulcerative process." 364.

*Treatment of the Ulcer of Bubo.* When granulations have sprung up, the ulcer is to be dressed in accordance with the principles which should regulate the dressings of all ulcers. On these we shall be silent. The resumption of mercury will hinge on these considerations:—the quantity the patient has already taken—the condition of the system, quoad the venereal virus, shewn by the state of the primary sore or its cicatrix, or by other circumstances—the state of the ulcerated bubo itself.

The continuance of the mercury, supposing it resumed, will be regulated by the general principles which we have pointed out elsewhere, and by a vigilant watch of the state of the ulcerated bubo itself. If the sore wears at all an irritable aspect, if the granulations are absorbed or do not advance, the surgeon will do well to consider whether he must not withhold the mercury again—whether he is giving too little, which seldom happens—or whether he is giving too much, which often happens. We know of few venereal conditions, excepting, perhaps, the venereal phagedæna, which require more caution, not to say skill, than the management of some ulcerated buboes. We shall return to this subject immediately. In the mean time, we pass to another, practically not unimportant.

A. It is the influence which the state of the primary symptom should exercise over the treatment of bubo. Mr. Wallace is the only author who touches upon this point. His observations on it are brief.

When a bubo, he says, and a primary symptom occur together, it unfortunately sometimes happens, that the indications presented by the former will be at variance with those presented by the latter. Thus a primary symptom which should not be treated with mercury may exist, along with a bubo which imperiously requires this remedy; or, a bubo may exist in a state opposed to the use of mercury, yet combined with a primary symptom requiring it. For example: an ulcer in its inflamed state, or during its ulcerating stage, and consequently when it might be injured by mercury, may possibly be accompanied by a bubo which might be resolved by this medicine. Again: the process of suppuration may be so far advanced in a bubo as to leave no hope of resolution from mercury, or the presence of that of ulceration may render the use of this medicine improper; while the primary ulcer is in the stage of reparation, and therefore peculiarly suited to mercurial treatment, &c. &c.

Such complicated or compound cases are always embarrassing, and their treatment must be left to the discretion of the practitioner; who should adopt, after mature consideration, that which promises, on the whole, the greatest advantage to his patient.

It is quite true, that occasional embarrassment ensues from the contradictory indications presented by the two symptoms. Yet we do not think the rule of practice in these cases so dubious as Mr. Wallace esteems it. That rule is never, if we can help it, to do harm. Injury is positive—good too often negative. The difficulty hinges on the administration of mercury. If this aggravates either the bubo or the sore, it should be discontinued; for, if persisted in, it may do great mischief, while a little delay in its administration for the symptom with which it does agree, can scarcely be of material consequence.

B. *Herpetic, or obstinate Ulcerations from Bubo.* We observed a short way back, that we should return to the subject of ulcerated bubo. We do so for the purpose of displaying some of the more troublesome, and, happily, more uncommon forms.

Mr. Hunter indulges in some rather obscure reasoning upon these cases. It does not amount to much, nor explain much. He suspects that when the ulcer takes on the anomalous conditions we shall exhibit presently, it is owing to some peculiarity in the constitution or the part. Probably this is, in general, the correct view of the case. But occasionally, the irritable state of the sore is clearly traceable to the improper use of mercury, which may have been given either at an improper time, or in improper quantities. It is clear, however, that the only mode of generalizing on exceptions, which such cases are, is to collect them, and to observe if they are reducible to any single formula. We shall quote some of the cases that we find in the pages of Mr. Hunter and of Dr. Colles.

CASE 1. "A gentleman had a very large venereal bubo, which was opened. He took a good deal of mercury for about two months; but, I suspect, not in sufficient doses, which produced a mercurial habit. The bubo had no disposition to heal, and I was consulted. From the account he gave me, I suspected that he had then too much of a mercurial habit to receive at this time any further good from that medicine. I therefore advised him to use a good nourishing diet for near a month; after that I put him upon a brisk mercurial course by friction; and the parts put on a better appearance. This course he continued for near two months, and then the sore, although much mended, began to be stationary. I did now conceive that the venereal action was destroyed, and therefore immediately left off the mercurial course, and put him upon a milk diet, and sent him into the country. But not gaining much ground, he had a strong decoction of the sarsaparilla with mezereon given him, which, although continued for above a month, produced little or no effect. I also gave him the cicuta as much as he could bear, with the bark almost the whole time, without effect: new sinuses formed, which were opened, and the sore became extremely irritable, with thickened lips. The dressings were poultices made with the juice of hemlock, sea-water, opium, and a gentle solution of lunar caustic; but nothing seemed to affect it. I suspected scrofula, and therefore proposed he should bathe in the sea; but this then could not be done. These different treatments, after mercury had been left off, took up about four months without the least benefit. Being doubtful whether there might not be still something venereal in the sore, especially as appearances were growing worse, and it was now four months since he had taken any mercury, I was inclined to try it once more, and sent him two portions of ointment, half an ounce each, to rub in in two nights. He had caught a little cold, and therefore did not rub in the mercury the two evenings as ordered; and called upon me the third day and told me he was much better. The sore now became easy, the watery or transparent inflammation began to subside, the lips became flatter and thinner, and the edges of the sore began to heal. I then desired him not to rub in the ointment, but wait a little. In eight or ten days the sore had contracted to three-quarters of its former size, and had all the appearance of a healing sore." 377.

This case proves little save this—that when such do well, we should be careful how in our own minds we attribute very much to our remedy or remedies. We will not comment on the dangerous doctrine of Mr. Hunter, that, if mercury be given for a lengthened period, a "mercurial habit" may be set up, *because* the mercury is not given in sufficient doses. The danger of the doctrine is only equalled by its ambiguity.

CASE 2. A gentleman had a common severe gonorrhœa. Mr. Hunter ordered him an injection of the corrosive sublimate, with a few mercurial pills. At the end of ten or twelve days he was no better, and Mr. Hunter desired him to be quiet. About this time a swelling took place in each groin, and Mr. Hunter, supposing them venereal, ordered mercurial inunction. The buboes suppurated, and while they were doing so, the gonorrhœa got well. The frictions were left off.

"The skin covering the buboes became thin; they were both opened, one with a caustic, the other with a lancet; he then was ordered to rub in mercury again on the thighs and legs for their cure. They began soon to look well, and to close fast; but when about half healed they became stationary. I suspected that a new disease was forming. On continuing the frictions a little longer they began to inflame and swell anew, and a suppuration took place about half an inch above each of the first suppurations, which broke into the first. I left off the mercury immediately upon their inflaming, and said that now a new disease had formed. I ordered poultices made with sea-water to be applied, and also a decoction of sarsaparilla to be taken; but this appeared not to be sufficient for the cure of this new disease. I then ordered him to go into the tepid sea-bath every evening, the heat of the water to be about ninety degrees. By the time he had been in the bath four times the inflammation and swelling had very much abated, and the first sores, or original buboes, were beginning to heal. He went on with the bathing every evening for about three weeks, when the sores rather began to look worse; I then suspected that the venereal disposition was become predominant, and I ordered the friction as before. In about a fortnight the first buboes healed, but the second suppurations were not yet healed; then I supposed it to be entirely the new-formed disease, and he went into the country, where I desired he might go into the open sea every day, as he then could have an opportunity, which he did, and got perfectly well, and has continued so." 378.

With all deference to Mr. Hunter, it seems highly probable that it was the treatment he adopted, which gave the case its severity. It is by no means clear, that mercury was ever required, nor is it clear that it was not productive of mischief. It is rather curious to watch Mr. Hunter's speculative reasoning and to note the manner in which he makes alternately one disease and another disease take possession of the sore.

Case 3. Mr. H. relates another case, in which the ulcerations spread in all directions, rising above the pubes, almost to the navel, and descending upon each thigh. A great variety of medicines were tried, particularly mercury in different forms, with little or no effect. Extract of hemlock did more good than anything else, and was taken in unusual quantities. 'An ounce was swallowed in the course of the day for some time, which was afterwards increased to an ounce and a half, two ounces, and even two ounces and a half. It produced indistinct vision and blindness, loss of the voice, falling of the lower jaw, a temporary palsy of the extremities, and once or twice a loss of sensation; and notwithstanding he was almost every night in a state, as it were, of complete intoxication from the hemlock, his general health did not suffer, but, on the contrary, kept pace in its improvement with the ulcers. After more than three years, the ulcers were nearly healed, when, having committed some irregularities in diet, the sores grew worse, and he returned to the extract of hemlock, which he had for some time laid aside, and of himself, swallowed in the course of the morning ten drachms. This quantity was only the half of what he had formerly taken in twenty-four hours, but his constitution had been at that time gradually habituated to the medicine. The ten drachms produced great restlessness and anxiety; he dropt insensible from his chair, fell into convulsions, and expired in two hours.

Dr. Colles alludes to the herpetic or creeping form of ulcerated bubo, under the appellation of the horse-shoe ulcer. He relates a case, of which we shall present the principal particulars.

Case 4. In 1823, Mr. H—, aged 30, contracted a sore on the frænum. Argentum nitratum was rubbed on the ulcer, and he used mercurial ointment under the care of a physician who made him repeatedly suspend the use of mercury the moment his mouth became ever so slightly affected.

Before he had ceased the course of frictions, a bubo appeared in his groin—this was punctured with a lancet. In five months after the appearance of the chancre he consulted an eminent surgeon in Cork, who prescribed some form of mercurial pills which slightly salivated him: he used only twelve of these. This surgeon then told him he should not use any more mercury, and advised sarsaparilla, lotions, and ointments of various kinds. Twelve months from the commencement of the disease he again consulted the same surgeon in consultation with Mr. Evans, the author of the Treatise on Ulcerations of the Genital Organs. Repeated venesections and low diet, (the results of their deliberations,) he states, induced great weakness, and nearly brought him to death's door.

When recovered from the debilitating effects of this plan, he repaired to London, where he remained for one month under the care of the late Mr. Abernethy. Blue pill every second night, and a sort of blue wash constituted the plan of treatment.

He next applied to another very eminent surgeon in London, who tried blue pill for a short time, but laid it aside because he thought it caused erysipelatous inflammation, and he cautioned him *never again* to use mercury. Under this gentleman's care he remained for two months trying a great variety of external applications, never employing the same beyond four or five days. He also used a variety of internal remedies, among which were acids, bark, and arsenic.

He next applied to the late Mr. John Pearson, and during six weeks that he remained under his care, he took sarsaparilla-syrup, and used a variety of local applications, of which he can only name carrot poultice.

In the Summer of that year he drank the Harrogate waters for one month, having been told that the sores were prevented from healing by a scorbutic state of his blood. He derived no benefit from these waters.

In the latter end of 1825, and 1826, he underwent long and repeated courses of sarsaparilla.

In October, 1826, he applied to Dr. Colles. The following was his condition at that time.

He has an ulcer, the size of a shilling, on the left outer ancle—this does not possess the character of a secondary venereal ulcer. In the left groin is a cicatrix, which beginning about the anterior spine of the ilium, is continued down the groin and passes to the back of the thigh, where it joins a prodigiously extensive ulcer; this ulcer reaches from the anus down along one-third of the back of the thigh; below the fold of the buttock it covers the entire breadth of the posterior part of the thigh. Above the fold of the buttock it is less wide, being in one place as narrow as one inch and a half. It is surrounded by a very deep edging of skin, which is cut very irregularly into knobs. The surface of this extensive ulcer is every where devoid of granulations, and presents three spots of deeper ulceration, in parts remote from each other. The entire of this ulcer is exquisitely sensitive and sore. Another branch of the cicatrix extends along the front of the thigh, and having traversed the upper third of the limb, it there ends in an irregular deep ulcer, the size of half-a-crown. The skin of the forehead near the roots of the hair is a deep red or copper colour, and the scalp is in a very scruffy condition. The general health is apparently very good. For three years he has been prevented by this disease from attending to any business.

Dr. Colles put him on mercurial inunction in half-scruple, and scruple doses. The sores were dressed with ointments containing mercury. On the 5th of November, the mouth was a little affected. On the 13th, the ulcers were all healing, but partially spreading. The dressings that agreed were cerati calaminæ ℥j. unguenti æruginis æris ʒij. M. On the 22nd, he was ordered sulphate of quinine. On the 7th of December, we find the following report:—He has now rubbed for twenty-four successive nights ung. hyd. fort. ℈i. by which his mouth is affected, but this affection does not increase according to the increased number of rubbings. The two small ulcers have scabbed; the large ulcer is very much reduced in extent, is much less irritable, and its edges are very healthy. Omitt. ung.—repet. sulph. quinæ.

The patient went on improving till the 28th. Then the quinine was omitted, and the mercurial ointment (℈ss. o. n.) resumed. This plan, with an addition to the quantity of mercury, was pursued till the 19th of January, when the ulcer had become irritable, and lost granulations, and the mouth was slightly affected. The mercurial ointment was omitted and the quinine resumed. From this moment, the ulcer improved uniformly, and, on the third of March, the patient returned to the country, with the ulcer on the very point of being perfectly healed.

We quite agree with Dr. Colles that, probably, this patient had a certain amount of the venereal virus lurking in his constitution. The *early* history and the consequences of the *latter* treatment both conspire to make this likely. Why it should have resisted mercury before, it would be unprofitable to enquire now. The mode of exhibition may not have been appropriate, or the state of constitution may not have been adapted for it. But the very evidence that proves mercury to have been serviceable in this case, proves small quantities only to have been useful. From the 28th of December it as palpably disagreed, as before that time it agreed with the sore.

We are not disposed to go into the treatment of the phagedænic, herpetic, or simply obstinate forms of ulcerated bubo at present, as that is involved in the wider question of the treatment of the phagedænic primary and secondary symptoms in general. We shall content ourselves with observing that there is no plan of management applicable to all cases. Small doses of mercury may be suited to one, will be destructive to many—tonics will be required in many cases—sedatives in most. There is nothing which more heavily taxes the judgment, experience, and caution of the surgeon than the treatment of phagedæna.

c. *Sinus between the Scrotum and Thigh.* This is another consequence of ulcerated bubo. It leads down from the pubic corner of the ulcer. We observe the skin, says Dr. Colles, in this situation to be red and raised; by pressing on this, we force out a little matter into the ulcer; from day to day we can see this sinus increasing in length. This disease teazes the patient for a long time, as it continues even after the accompanying chancre has healed, under the influence of mercury. Pressure has no influence in arresting the progress, or in effecting the cure of this sinus. The treatment to be pursued in this case is either to leave it to the restorative powers of the system, or if these prove inefficient, we may divide it in its entire length, or make a small opening into it inferiorly, and daily inject some stimulant fluid.

This is Dr. Colles' recommendation. The general state of the patient being properly attended to, the best mode of treating this sinus is by dividing it—the next best, by passing a seton through it—the worst (so we have found it) by the injection.

D. *Indolent Syphilitic Bubo.*—Mr. Wallace devotes some pages to the consideration of this form of bubo. Its peculiarities are, according to that author, the following:—1st. More glands than one are generally enlarged in the disease. 2d. The surrounding cellular substance is extensively affected. 3d. The integuments, when engaged in the disease, are of a deep livid or purple red. 4th. The matter slowly approaches to the skin by one extensive surface, or at successive times by a number of smaller surfaces. In both cases, the vitality of a large portion of integument is often destroyed, or its texture greatly altered, from whence there results, either an ulcer with undermined edges and detached flaps, or else numerous fistulous openings. 5th. The matter discharged deviates, more or less, in its characters, from thick homogenous whitish-yellow pus,—being serous and sanguineous, or serous and curd-like, or a thickish glairy or viscid fluid. And *finally*, the processes are slow, and attended with a febrile condition of system.

It is commonly supposed that buboes which display this peculiarly sluggish state are scrofulous. We agree with Mr. Wallace that the term is much abused. That the cause of the peculiarity is in the constitution is most probable—that there is any single cause operating in all cases is not probable. Each case must be investigated and treated in a special manner. Some general rules may assist us in the management of the particular case.

1. When there is pyrexia, mild antiphlogistic remedies are indicated. The secretions may be improved, and evaporating lotions, or poultices applied according to circumstances. Leeches, too, *may* be useful.

2. When pyrexia is removed, mercury may be gently tried. But it must be tried as an experiment, and a very cautious experiment too.

3. Tonics are certainly necessary. Sometimes one, sometimes another answers best. Sarsaparilla with the liquor potassæ usually agrees. Occasionally the mineral acids are useful. Commonly it is necessary to change the medicines frequently.

4. Blisters are often of service, previously to suppuration.

5. A moderately early opening should be made, if there is matter. Mr. Wallace recommends the potassa fusa if the skin is past hope of recovery.

6. When the ulcer is formed, it must be treated in accordance with general principles. It would be useless going into details. Change of air—the sea-side—every thing to invigorate, become more requisite than ever.

7. Mr. Wallace speaks highly of a mode of applying the nitrate of silver in these cases. If, he says, there be reason to suppose, as very frequently happens, that the diseased integuments can be saved, we are to proceed in an entirely different manner from that just mentioned. We should rub the nitrate of silver on the surface of the bubo, and of the surrounding diseased skin, previously moistened with tepid water, until the cuticle is rendered of a bluish colour to the extent of an inch beyond the diseased integuments covering the tumour. This application is adapted not only for the bubo prior to the discharge of pus, but even to the unhealthy integument around the ulcer.

This concludes what we have to say at present on the management of syphilitic bubo. If our younger readers receive any clearer notions on the subject we shall be amply satisfied.

